# Factors associated with facility-based delivery in Mayoyao, Ifugao Province, Philippines

**DOI:** 10.1186/1447-056X-12-5

**Published:** 2013-10-24

**Authors:** Azusa Shimazaki, Sumihisa Honda, Marcelyn M Dulnuan, Jennylene B Chunanon, Akiko Matsuyama

**Affiliations:** 1 Department of Nursing, School of Health Sciences, Nagasaki University, Nagasaki, Japan; 2 Department of Community-based Rehabilitation Sciences, Graduate School of Biomedical Sciences, Nagasaki University, Nagasaki, Japan; 3 JICA-MCH Project, Ifugao, Philippines; 4 Mayoyao Municipal Health Office, Ifugao, Philippines; 5 Graduate School of International Health Development, Nagasaki University, 1-12-4, Sakamoto, Nagasaki 852-8523, Japan

**Keywords:** Maternal health, Delivery location, Decision making, Women’s autonomy

## Abstract

**Background:**

The maternal mortality ratio (MMR) in the Philippines is higher than in most other Southeast Asian countries, and home delivery is a major factor contributing to the high MMR. This study aims to explore the determinants for choice of delivery location in Ifugao Province, where people have poor access to health services.

**Findings:**

A household interview survey using a structured questionnaire was conducted to identify the factors associated with delivery location among 354 women. In all, 44.4% of the respondents delivered at a health facility. Using logistic regression analysis, parity (odds ratio [OR] 3.0, 95% confidence interval [C.I.] 1.6-5.6), higher education (OR 5.9, 95% C.I. 2.7-12.9), distance to a health facility (OR 6.9, 95% C.I. 3.4-14.2), health problems identified at antenatal care (OR 2.4, 95% C.I. 1.3-4.6), and the person deciding on the delivery location (e.g., for the husband OR 3.2, 95% C.I. 1.1-9.4) were found to be statistically associated with facility-based delivery.

**Conclusion:**

Involving the husband and other people in the decision regarding delivery location may influence a woman’s choice to use facility-based delivery services. Our findings have useful implications for improving the existing Safe Motherhood program in the Philippines.

## Findings

### Background

Every year, 536,000 maternal deaths occur worldwide, and 99% of these occur in developing countries [1]. Most maternal deaths occur because of delays in obtaining adequate medical care. Delays in obtaining obstetric emergency care have been identified as comprising three types: (1) delay in the decision to seek care; (2) delay in arrival at a health facility; and (3) delay in the provision of adequate care [2]. To reduce maternal deaths, the most efficient strategy for lower-income countries is to promote childbirth at health facilities with a referral capacity [3-5].

Although a policy for promoting facility-based delivery has been introduced by the government of the Philippines [6], the maternal mortality rate (MMR) was 230 per 100,000 live births in 2008, still higher than in most other Southeast Asian countries [7]. Such a high MMR is largely attributable to the fact that 56% of women in the Philippines delivered at home [8]. From 2006 to 2010, the Japan International Cooperation Agency (JICA) implemented a health project directed at maternal and childcare practices, whereby facility-based delivery was promoted in two representative provinces in the Philippines, Billiran and Ifugao. In Billiran, the proportion of facility-based deliveries increased dramatically from 30% in 2005 to 89% in 2009. However, the response was less marked in Ifugao where facility-based deliveries increased from 19% to 34% over the same period [9].

We conducted an exploratory study in Ifugao to determine sociodemographic characteristics and details of delivery location, and to explore the factors affecting the use of facility-based delivery services from the women’s viewpoint.

## Methods

### Study area

The study was conducted in the municipality of Mayoyao, Ifugao Province, in northern Luzon Island. Mayoyao is 2,000 km from Manila, and its population was 16,990 in 2006. Rugged mountains characterize the topography, with elevations ranging from 400 to 1,800 meters above sea level. Agriculture, especially rice cultivation, is the major occupation in the area. Markets, high schools, and medical facilities are located in the central part of Mayoyao, which is a maximum of 1 day’s travel on foot or by motorcycle from the most remote villages.

The municipality consists of 27 villages called *barangays*. A district hospital and a municipal health office, which functions as a rural health unit (RHU), are located in the central area. A barangay health station (BHS) is located in every two to four villages, and in total, eight BHSs are in operation in the municipality. A midwife is allocated to each BHS, except for the five barangays in the central part of the municipality, which are under the jurisdiction of the RHU. Taking accessibility issues into account, 15 barangays, serviced by the RHU and three BHSs, were selected for the study.

### Study participants

The names of 388 women who gave birth from August 2006 to July 2009 were listed in the birth registry of the Mayoyao Municipal Health Office. Thirty-four women were excluded because either they delivered their babies outside Ifugao or they were absent during the research period. Therefore, the study included 354 women. The women’s health team, including local traditional birth attendants (TBAs) and midwives organized by the JICA project had endeavored to identify all pregnant women in the area, so not only those who delivered at health facilities but also women who delivered at home were theoretically included in the registry.

### Data collection and analysis

A household interview survey using a structured questionnaire was conducted from September to November 2009 for all 354 women listed in the birth registry. Eight data collectors were hired and trained by one of the authors. During their 3-day training period, the data collectors translated the questionnaire from English into the local languages of Majawjaw and Ilocano. Respondents were asked about their sociodemographic background, their household income and assets (including possession of radios, televisions, cellular phones, refrigerators, and motorcycles), about their perceptions of delivery risk and maternal death, and their care-seeking behavior during pregnancy and childbirth (including the type of birth attendant, delivery location, antenatal care visits, and who made the decision regarding delivery location).

Categorical data were analyzed using chi-square and Cochran-Armitage tests. Multiple logistic regression analysis was performed using Akaike’s information criteria to identify factors affecting the delivery location. SPSS version 17.0 (SPSS Japan Inc., Tokyo, Japan) was used.

The ethical committee of the Graduate School of International Health Development, Nagasaki University, approved the research proposal and the Ifugao Provincial Health Office also granted permission for the research. All respondents were asked to provide written informed consent prior to taking part in the interviews.

## Results

### Baseline characteristics

The characteristics of the women by delivery location are shown in Table 1. Of the respondents, 55.6% delivered at home and 44.4% delivered at a facility. There were significantly more multiparas in the home-delivery group than in the facility-delivery group (p < 0.001). Women who delivered at home were significantly more likely to have larger families (p = 0.046), to be economically worse off (p < 0.001), to need a longer time to travel to the nearest birthing facility (p < 0.001), to have lower household asset scores (p < 0.001), and to have lower education levels (p < 0.001). The associations between antenatal care-related issues and delivery location are shown in Table 2. Health problems were discovered more frequently during antenatal care among women who delivered at a facility (p < 0.001). Women who delivered at a facility were more likely to involve their husbands and other people in the decision about delivery location (p < 0.001) (Table 3).

**Table 1:** Women’s sociodemographic factors and characteristics by delivery location

	**Home-delivery group**	**Facility-delivery group**	
	**(n = 197)**	(n = 157*)	
**Age of woman (years)**			
≤25	45 (23.3%)	54 (34.6%)	
26-30	54 (28.0%)	29 (18.6%)	
≥31	94 (48.7%)	73 (46.8%)	
Unknown	4	1	p = 0.150^a^
**Parity**			
Primipara	27 (13.7%)	60 (38.2%)	p < 0.001^b^
Multipara	170 (86.3%)	97 (61.8%)	
**No. of family members**			
1-4	53 (26.9%)	56 (35.7%)	
5-7	93 (47.2%)	71 (45.2%)	
≥8	51 (25.9%)	30 (19.1%)	p = 0.046^a^
**Monthly income per capital**			
≤500 pesos	118 (59.9%)	44 (28.0%)	
501-1,000 pesos	41 (20.8%)	36 (22.9%)	
≥1,000 pesos	38 (19.3%)	77 (49.0%)	p < 0.001^a^
**Household asset score**^**§**^			
≤2	76 (50.7%)	28 (18.3%)	
3-4	49 (32.7%)	42 (27.5%)	
≥5	25 (16.7%)	83 (54.2%)	
Unknown	47	4	p < 0.001^a^
**Education level**			
Elementary or lower	67 (34.0%)	14 (9.0%)	
High school	73 (37.1%)	38 (24.4%)	
Post-high school	57 (28.9%)	104 (66.7%)	
Unknown	0	1	p < 0.001^a^
**Time required to reach the nearest birthing facility**			
≤10 min	38 (19.4%)	79 (50.3%)	
11-30 min	65 (33.2%)	57 (36.3%)	
≥31 min	93 (47.4%)	21 (13.4%)	
Unknown	1	3	p < 0.001^a^

^a^Cochran-Armitage test. ^b^Chi-square test. *Including 144 women who delivered at a hospital, six women who delivered at a RHU/BHS, and seven women who delivered at other facilities, mainly Ifugao provincial hospital.

^§^Household asset score was calculated by summing each household asset item including radios, televisions, cellular phones, refrigerators, and motorcycles. These items were selected from Philippines Demographic and Health Survey [8].

**Table 2:** Antenatal care-related issues by delivery location

	**Home-delivery group**	**Facility-delivery group**	
	**(n = 197)**	**(n = 157)**	
**Antenatal care visit**			
At least once	190 (96.4%)	157 (100.0%)	p = 0.016^a^
Never	7 (3.6%)	0 (0.0%)	
**Health problem found at antenatal care**^**b**^			
Yes	32 (16.8%)	56 (35.7%)	p < 0.001^a^
No	158 (83.2%)	101 (64.3%)	
**Experienced health problems during pregnancy**			
Yes	124 (63.3%)	105 (66.9%)	p = 0.480^a^
No	72 (36.7%)	52 (33.1%)	
Unknown	1	0	

^a^Chi-square test. ^b^This question was asked only if the woman had received antenatal care.

**Table 3:** Decision maker on delivery location

	**Home-delivery group**	**Facility-delivery group**	
	**(n = 197)**	**(n = 157)**	
Only myself	188 (95.9%)	119 (79.9%)	p < 0.001^a^
Mainly husband	6 (3.1%)	19 (12.8%)	
Mainly others	2 (1.0%)	11 (7.4%)	
Unknown	1	8	

^a^Cochran-Armitage test.

### Supportive factors for facility-based delivery

Five variables were selected for logistic regression using Akaike’s information criteria. These independent variables were parity, educational level, time required to reach the nearest birthing facility, decision maker about delivery location, and health problems found during antenatal care. The results are presented in Table 4.

**Table 4:** Logistic regression analysis of the association between facility delivery and several variables

**Variable**		**Odds ratio**	**95% C.I.**
Parity	Multipara	1	
	Primipara	3.0	1.6-5.6
Education level	Less than primary school	1	
	Secondary school (High school)	2.1	0.9-4.9
	Higher than secondary school	5.9	2.7-12.9
Time required to reach the nearest birthing facility	≥31 min	1	
	11-30 min	3.3	1.7-6.6
	≤10 min	6.9	3.4-14.2
Decision maker on location of delivery	Myself	1	
	Husband	3.2	1.1-9.4
	Others	6.0	1.1-31.3
Health problem found at antenatal care	No	1	
	Yes	2.4	1.3-4.6

## Discussion

Our results reveal that involving family members, such as their husband, or other community members, in the decision-making process is important for women when seeking facility-delivery services. Previous studies on the relationship between reproductive health-seeking behavior and women’s autonomy have yielded mixed results. Women with greater autonomy generally have an increased ability to make favorable reproductive health decisions, particularly in South Asia [10-13]. However, this was not the case in a study in Kenya [14]. The social status of women in the study area is generally high, as it is elsewhere in the Philippines [15], and the *bayanihan* spirit of mutual help is an old and very strong value [16]. The data in Table 3 indicate that 80% of women in the facility-delivery group made the decision on where to give birth alone compared with 96% in the home-delivery group. In this study, the results may have reflected not only women’s autonomy per se but also the level of support and shared understanding of their husbands and other community members in the decision regarding facility-based delivery. Unfortunately, we did not clearly define others in the decision maker question, and additional factors involved in the decision-making process were not explored further.

Parity is a strong predictor of delivery location, as indicated in a number of previous studies [17-19]. The health staff in this study tended to recommend that women should deliver at a facility, at least for the first childbirth, which may have affected the results. The educational level of women as an important predictor of health-service use has been documented elsewhere [20-23]. In Mayoyao, most information sources are not in the local dialect, Majawjaw, but in the national language, Tagalog, or English. Less-educated individuals sometimes need to be assisted by health staff in reading health educational materials. Geographic accessibility is another determinant of maternal care-seeking behavior [2,24].

There were some limitations to the present study. First, the study site, which occupies the central part of a local municipality, is geographically more advantageous than remote mountainous areas, and more women tend to go to hospital rather than to the RHU or a BHS for delivery. Therefore, the study results cannot be generalized to all regions. Second, although skilled birth attendants (SBAs) assisted women with home delivery in some cases (27 of 197), these women were included in the home-delivery group. We performed an analysis of the relationship between the type of birth attendant, SBAs, including those who attended at home, versus non-SBAs (TBAs), and a number of factors. The results were identical to those for delivery location indicating that the type of birth attendant did not alter the outcome (data not shown). Third, this study was analyzed based on the responses from women only, and their husbands’ perception was not examined.

## Conclusions

In Mayoyao, one-third of pregnant women selected a facility-based delivery. Involvement of the husband and other support people in the decision regarding delivery location is essential in promoting facility-based delivery. Husbands and other community members should be empowered with substantial action plans, including preparation for obstetric emergencies. In addition, further studies to address gaps in the research, particularly with regard to the perceptions of both men and women on women’s reproductive health issues, are essential.

## Competing interests

The authors declare that they have no competing interests.

## Authors’ contributions

AS worked on this research for her graduate degree program. MMD and JBC coordinated the field study. SH participated in the design of the study and performed the statistical analysis. AM proposed the concept of the study and helped to draft the manuscript. All authors read and approved the final manuscript.

## Authors’ information

AS, M.P.H., Nurse Midwife, preformed midwifery services for 2 years and worked for JICA technical cooperation projects in Sudan, Fiji, and Vanuatu.

## References

[B1] WHO, UNICEF, UNFPA, World Bank Maternal mortality in 2005 2007 Geneva: WHO, UNICEF,WHO,UNFPA, and the World Bank

[B2] Thaddeus S Maine D Too far to walk: maternal mortality in context Soc Sci Med 1994 12 8 1091 1110 10.1016/0277-9536(94)90226-7 8042057

[B3] Danforth E Kruk M Rockers P Mbaruku G Galea S Household decision-making about delivery in health facilities: evidence from Tanzania J Health Popul Nutr 2009 12 5 696 703 19902806 10.3329/jhpn.v27i5.3781 PMC2928090

[B4] Koblinsky M Matthews Z Hussein J Mavalankar D Mridha MK Anwar I Achadi E Adjei S Padmanabhan P Lerberghe WV Going to scale with professional skilled care Lancet 2006 12 9544 1377 1386 10.1016/S0140-6736(06)69382-3 17046470

[B5] WHO The world Health Report 2005 2005 Geneva: WHO Press

[B6] Philippines Department of Health Women's Health and Safe Motherhood Project[http://www.doh.gov.ph/node/1076.html]

[B7] UNICEF The state of the world’s children 2008 2008 New York: UNICEF

[B8] Philippines National Statistics Office and ORC Macro Philippines National Demographic and Health Survey 2003 Maryland: Philippines National Statistics Office and ORC Macro

[B9] JICA Annual Report 2008 2008 Manila: Maternal and Child Health Project

[B10] Mistry R Galal O Lu M Women’s Autonomy and pregnancy care in rural India: a contextual analysis Soc Sci Med 2009 12 6 926 933 10.1016/j.socscimed.2009.07.008 19656604

[B11] Schuler SR Hashemi SM Credit programs, women’s empowerment, and contraceptive use in rural Bangladesh Stud Fam Plann 1994 12 2 65 76 10.2307/2138085 8059447

[B12] Jejeebhoy S Women’s Education, autonomy, and reproductive behaviour: experience from developing countries 1995 Oxford: Clarendon

[B13] Upadhyay U Hindin M Do higher status and more autonomous women have longer birth intervals?: results from Cebu, Philippines Soc Sci Med 2005 12 11 2641 2655 10.1016/j.socscimed.2004.11.032 15814188

[B14] Fotso J Ezeh A Essendi H Maternal health in resource-poor urban settings: How does women’s autonomy influence the utilization of obstetric care services? Reprod Health 2009 12 1 9 10.1186/1742-4755-6-9 19531235 PMC2706794

[B15] Mason K Eugenia M How family position influences married women's autonomy and power in five Asian countries Meeting Report of Committee for International Cooperation in National Research in Demography: 24-26 February 1997; Paris 1997 Paris: East-West Center 353 371

[B16] Andres TD Understanding Ifugao values 2004 Manila: A Giraffe Book

[B17] Wagle RR Sabroe S Nielsen BB Socioeconomic and physical distance to the maternity hospital as predictors for place of delivery: an observation study from Nepal BMC Pregnancy Childbirth 2004 12 1 8 10.1186/1471-2393-4-8 15154970 PMC425583

[B18] Rogan SEB Olvena MVR Factors affecting maternal health utilizaiton in the Philippines9th National Convention on Statistics 2004 Manila: National Statistics Office

[B19] Gage AJ Barriers to the utilization of maternal health care in rural Mali Soc Sci Med 2007 12 8 1666 1682 10.1016/j.socscimed.2007.06.001 17643685

[B20] Nwakoby B Use of obstetric services in rural Nigeria J R Soc Health 1994 12 3 132 10.1177/146642409411400304 7932482

[B21] Raghupathy S Education and the use of maternal health care in Thailand Soc Sci Med 1996 12 4 459 471 10.1016/0277-9536(95)00411-4 8844947

[B22] Ikeako L Onah H Iloabachie G Influence of formal maternal education on the use of maternityservices in Enugu, Nigeria J Obstet Gynaecol 2006 12 1 30 34 10.1080/01443610500364004 16390706

[B23] Onah H Ikeako L Iloabachie G Factors associated with the use of maternity services in Enugu, southeastern Nigeria Soc Sci Med 2006 12 7 1870 1878 10.1016/j.socscimed.2006.04.019 16766107

[B24] Gabrysch S Campbell OM Still too far to walk: literature review of the determinants of delivery service use BMC Pregnancy Childbirth 2009 12 34 10.1186/1471-2393-9-34 19671156 PMC2744662

